# Supplementary material for: Pathogens and Antibiotic Susceptibilities of Global Bacterial Keratitis: A Meta-Analysis
Source: Antibiotics (Basel). 2022 Feb 12;11(2):238. doi: 10.3390/antibiotics11020238 (PMC8868051; doi:10.3390/antibiotics11020238)
Supplement: Supplementary file 1 [file antibiotics-11-00238-s001.zip › antibiotics-1584882-supplementary.pdf]

antibiotics

MDPI

Article

Pathogens and Antibiotic Susceptibilities of Global Bacterial Keratitis: A Meta-Analysis

Zijun Zhang, Kai Cao, Jiamin Liu, Zhenyu Wei, Xizhan Xu and Qingfeng Liang *

Beijing Institute of Ophthalmology, Beijing Tongren Eye Center, Beijing Tongren Hospital, Capital Medical University, Beijing Key Laboratory of Ophthalmology and Visual Sciences, Beijing 100005, China; shenyu@ccmu.edu.cn (Z.Z.); caozhi@ccmu.edu.cn (K.C.); liujiamin@mail.ccmu.edu.cn (J.L.); weizhenyu@ccmu.edu.cn (Z.W.); xuxz0924@mail.ccmu.edu.cn (X.X.)

* Correspondence: lqflucky@163.com

Supplementary Materials:

Table S1. Positive rate of bacterial culture from corneal lesions in different regions.

	Sample size	Positive rate	95%CI	Extrapolated from
**Europe**	8353	42%	[34%; 50%]	[1-10]
Ireland	90	36%	[26%; 46%]	[3]
UK	7437	40%	[32%; 48%]	[4, 6, 7, 9]
Germany	346	43%	[38%; 48%]	[5]
Portugal	183	31%	[25%; 38%]	[8]
Spain	297	65%	[59%; 70%]	[10]
**Africa**	73	58%	[46%; 68%]	[11]
Sierra Leone	73	58%	[46%; 68%]	[11]
**Asia**	12106	42%	[33%; 51%]	[12-27]
India	4417	44%	[31%; 58%]	[13, 19, 23, 24]
Turkey	620	28%	[25%; 32%]	[14]
Nepal	458	39%	[35%; 43%]	[15-17]
Korea	129	83%	[75%; 88%]	[26]
Malaysia	189	30%	[24%; 37%]	[27]
China	5350	41%	[28%; 55%]	[20, 22, 25]
Israel	943	44%	[41%; 47%]	[21]
**Oceania**	511	59%	[48%; 68%]	[28-31]
New Zealand	352	63%	[58%; 68%]	[28, 31]
Australia	159	55%	[35%; 74%]	[29, 30]
**America**	17888	51%	[41%; 62%]	[32-43]
Canada	4312	60%	[47%; 72%]	[35, 41, 43]
USA	11938	51%	[37%; 64%]	[32-34, 36, 38-40, 42]
Mexico	1638	33%	[31%; 36%]	[37]

References

1. Schaefer, F., O. Bruttin, L. Zografos, et al., *Bacterial keratitis: a prospective clinical and microbiological study.* Br J Ophthalmol 2001. **85**, 842-7.
2. Bourcier, T., F. Thomas, V. Borderie, et al., *Bacterial keratitis: predisposing factors, clinical and microbiological review of 300 cases.* Br J Ophthalmol 2003. **87**, 834-8.
3. Saeed, A., F. D'Arcy, J. Stack, et al., *Risk factors, microbiological findings, and clinical outcomes in cases of microbial keratitis admitted to a tertiary referral center in ireland.* Cornea 2009. **28**, 285-92.
4. Orlans, H.O., S.J. Hornby, and I.C. Bowler, *In vitro antibiotic susceptibility patterns of bacterial keratitis isolates in Oxford, UK: a 10-year review.* Eye (Lond) 2011. **25**, 489-93.

5. Prokosch, V., Z. Gatzioufas, S. Thanos, et al., *Microbiological findings and predisposing risk factors in corneal ulcers.* Graefes Arch Clin Exp Ophthalmol 2012. **250**, 369-74.
6. Otri, A.M., U. Fares, M.A. Al-Aqaba, et al., *Profile of sight-threatening infectious keratitis: a prospective study.* Acta Ophthalmol 2013. **91**, 643-51.
7. Tan, S.Z., A. Walkden, L. Au, et al., *Twelve-year analysis of microbial keratitis trends at a UK tertiary hospital.* Eye (Lond) 2017. **31**, 1229-1236.
8. Ferreira, C.S., L. Figueira, N. Moreira-Gonçalves, et al., *Clinical and Microbiological Profile of Bacterial Microbial Keratitis in a Portuguese Tertiary Referral Center-Where Are We in 2015?* Eye Contact Lens 2018. **44**, 15-20.
9. Tavassoli, S., G. Nayar, K. Darcy, et al., *An 11-year analysis of microbial keratitis in the South West of England using brain-heart infusion broth.* Eye (Lond) 2019. **33**, 1619-1625.
10. Tena, D., N. Rodríguez, L. Toribio, et al., *Infectious Keratitis: Microbiological Review of 297 Cases.* Jpn J Infect Dis 2019. **72**, 121-123.
11. Capriotti, J.A., J.S. Pelletier, M. Shah, et al., *The etiology of infectious corneal ulceration in Sierra Leone.* Int Ophthalmol 2010. **30**, 637-40.
12. Kunimoto, D.Y., S. Sharma, P. Garg, et al., *Corneal ulceration in the elderly in Hyderabad, south India.* Br J Ophthalmol 2000. **84**, 54-9.
13. Sharma, S., M. Taneja, R. Gupta, et al., *Comparison of clinical and microbiological profiles in smear-positive and smear-negative cases of suspected microbial keratitis.* Indian J Ophthalmol 2007. **55**, 21-5.
14. Yilmaz, S., I. Ozturk, and A. Maden, *Microbial keratitis in West Anatolia, Turkey: a retrospective review.* Int Ophthalmol 2007. **27**, 261-8.
15. Lavaju, P., S.K. Arya, B. Khanal, et al., *Demograhic pattern, clinical features and treatment outcome of patients with infective keratitis in the eastern region of Nepal.* Nepal J Ophthalmol 2009. **1**, 101-6.
16. Feilmeier, M.R., K.R. Sivaraman, M. Oliva, et al., *Etiologic diagnosis of corneal ulceration at a tertiary eye center in Kathmandu, Nepal.* Cornea 2010. **29**, 1380-5.
17. Dhakhwa, K., M.K. Sharma, S. Bajimaya, et al., *Causative organisms in microbial keratitis, their sensitivity pattern and treatment outcome in western Nepal.* Nepal J Ophthalmol 2012. **4**, 119-27.
18. Hong, J., J. Chen, X. Sun, et al., *Paediatric bacterial keratitis cases in Shanghai: microbiological profile, antibiotic susceptibility and visual outcomes.* Eye (Lond) 2012. **26**, 1571-8.
19. Aruljyothi, L., N. Radhakrishnan, V.N. Prajna, et al., *Clinical and microbiological study of paediatric infectious keratitis in South India: a 3-year study (2011-2013).* Br J Ophthalmol 2016. **100**, 1719-1723.
20. Hsiao, C.H., C.C. Sun, L.K. Yeh, et al., *Shifting Trends in Bacterial Keratitis in Taiwan: A 10-Year Review in a Tertiary-Care Hospital.* Cornea 2016. **35**, 313-7.
21. Politis, M., D. Wajnsztajn, B. Rosin, et al., *Trends of Bacterial Keratitis Culture Isolates in Jerusalem; a 13- Years Analysis.* PLoS One 2016. **11**, e0165223.
22. Lin, L., W. Lan, B. Lou, et al., *Genus Distribution of Bacteria and Fungi Associated with Keratitis in a Large Eye Center Located in Southern China.* Ophthalmic Epidemiol 2017. **24**, 90-96.
23. Bagga, B., S.R. Motukupally, and A. Mohamed, *Microbial keratitis in Stevens-Johnson syndrome: Clinical and microbiological profile.* Ocul Surf 2018. **16**, 454-457.
24. Das, S., R. Samantaray, A. Mallick, et al., *Types of organisms and in-vitro susceptibility of bacterial isolates from patients with microbial keratitis: A trend analysis of 8 years.* Indian J Ophthalmol 2019. **67**, 49-53.
25. Liu, H.Y., H.S. Chu, I.J. Wang, et al., *Microbial Keratitis in Taiwan: A 20-Year Update.* Am J Ophthalmol 2019. **205**, 74-81.
26. Mun, Y., M.K. Kim, and J.Y. Oh, *Ten-year analysis of microbiological profile and antibiotic sensitivity for bacterial keratitis in Korea.* PLoS One 2019. **14**, e0213103.
27. Khor, H.G., I. Cho, K. Lee, et al., *Spectrum of Microbial Keratitis Encountered in the Tropics.* Eye Contact Lens 2020. **46**, 17-23.
28. Hall, R.C. and M.J. McKellar, *Bacterial keratitis in Christchurch, New Zealand, 1997-2001.* Clin Exp Ophthalmol 2004. **32**, 478-81.
29. Ly, C.N., J.N. Pham, P.R. Badenoch, et al., *Bacteria commonly isolated from keratitis specimens retain antibiotic susceptibility to fluoroquinolones and gentamicin plus cephalothin.* Clin Exp Ophthalmol 2006. **34**, 44-50.
30. Constantinou, M., V. Jhanji, L.W. Tao, et al., *Clinical review of corneal ulcers resulting in evisceration and enucleation in elderly population.* Graefes Arch Clin Exp Ophthalmol 2009. **247**, 1389-93.
31. Pandita, A. and C. Murphy, *Microbial keratitis in Waikato, New Zealand.* Clin Exp Ophthalmol 2011. **39**, 393-7.
32. Alexandrakis, G., E.C. Alfonso, and D. Miller, *Shifting trends in bacterial keratitis in south Florida and emerging resistance to fluoroquinolones.* Ophthalmology 2000. **107**, 1497-502.
33. Yeh, D.L., S.S. Stinnett, and N.A. Afshari, *Analysis of bacterial cultures in infectious keratitis, 1997 to 2004.* Am J Ophthalmol 2006. **142**, 1066-8.
34. Afshari, N.A., J.J. Ma, S.M. Duncan, et al., *Trends in resistance to ciprofloxacin, cefazolin, and gentamicin in the treatment of bacterial keratitis.* J Ocul Pharmacol Ther 2008. **24**, 217-23.
35. Lichtinger, A., S.N. Yeung, P. Kim, et al., *Shifting trends in bacterial keratitis in Toronto: an 11-year review.* Ophthalmology 2012. **119**, 1785-90.
36. Lin, C.C., P. Lalitha, M. Srinivasan, et al., *Seasonal trends of microbial keratitis in South India.* Cornea 2012. **31**, 1123-7.
37. Hernandez-Camarena, J.C., E.O. Graue-Hernandez, M. Ortiz-Casas, et al., *Trends in Microbiological and Antibiotic Sensitivity Patterns in Infectious Keratitis: 10-Year Experience in Mexico City.* Cornea 2015. **34**, 778-85.

38. Sand, D., R. She, I.A. Shulman, et al., *Microbial keratitis in los angeles: the doheny eye institute and the los angeles county hospital experience.* Ophthalmology 2015. **122**, 918-24.
39. Jin, H., W.T. Parker, N.W. Law, et al., *Evolving risk factors and antibiotic sensitivity patterns for microbial keratitis at a large county hospital.* Br J Ophthalmol 2017. **101**, 1483-1487.
40. Rossetto, J.D., K.M. Cavuoto, C.J. Osigian, et al., *Paediatric infectious keratitis: a case series of 107 children presenting to a tertiary referral centre.* Br J Ophthalmol 2017. **101**, 1488-1492.
41. Tam, A.L.C., E. Côté, M. Saldanha, et al., *Bacterial Keratitis in Toronto: A 16-Year Review of the Microorganisms Isolated and the Resistance Patterns Observed.* Cornea 2017. **36**, 1528-1534.
42. Peng, M.Y., V. Cevallos, S.D. McLeod, et al., *Bacterial Keratitis: Isolated Organisms and Antibiotic Resistance Patterns in San Francisco.* Cornea 2018. **37**, 84-87.
43. Termote, K., A.W. Joe, A.L. Butler, et al., *Epidemiology of bacterial corneal ulcers at tertiary centres in Vancouver, B.C.* Can J Ophthalmol 2018. **53**, 330-336.
